# Species diversity patterns in *Tagetes minuta*-invaded plant communities along an elevational gradient in Southeastern Xizang

**DOI:** 10.7717/peerj.20573

**Published:** 2026-01-08

**Authors:** Norzin Tso, Ngawang Norbu, Wei Li, Xin Tan, Zhefei Zeng, La Qiong, Junwei Wang

**Affiliations:** 1 Key Laboratory of Biodiversity and Environment on the Qinghai-Tibetan Plateau, Ministry of Education, School of Ecology and Environment, Xizang University, Lhasa, China; 2 Yani Observation and Research Station for Wetland Ecosystem of the Xizang Autonomous Region, Xizang University, Nyingchi, China

**Keywords:** Invasive plants, *Tagetes minuta*, Habitat types, Species diversity, Elevation gradients

## Abstract

*Tagetes minuta*, a herbaceous plant native to South America, has shown a significant trend of invasion along the section from Nyingchi to Shannan, situated along the Yarlung Zangbo River in southeastern Xizang in recent years. In this study, we conducted field surveys of *T. minuta* plant communities at elevations ranging from 2,925 to 3,553 m. By establishing 31 quadrats, we systematically analyzed the species composition, diversity characteristics of the invaded communities of *T. minuta*, and their relationships with elevation gradients and habitat types. The study results revealed that a total of 78 plant species, belonging to 28 families and 69 genera, were recorded in the *T. minuta*-invaded plant communities. Among them, the families Asteraceae, Poaceae, and Rosaceae were dominant, with herbaceous plants being in an absolute majority. The diversity analysis showed that the Shannon–Wiener index, Simpson index, and Pielou’s evenness index of were significantly higher in the community group mainly composed of *Eragrotis pilosa* and *Plantago depressa* (Cluster Group II) compared to the groups dominated by *Poa annua* plus *Plantago depressa* (Cluster Group I) and *Poa annua* and *Digitaria cruciata* (Cluster group III) (*P* < 0.05), while no significant differences were found in species richness. This suggests that the invasion of *T. minuta* primarily affects the evenness of species distribution rather than species richness. In addition, the species diversity indices of the *T. minuta*-invaded plant communities showed no significant correlation with elevation, indicating that elevation is not a major factor influencing species diversity in the invaded communities. The height of *T. minuta* was significantly positively correlated with elevation (*P* < 0.01), while its cover showed no significant correlation with elevation. Under different habitat types, the height and cover of *T. minuta* showed significant differences, with stronger invasion ability in habitats with greater human disturbance. This study highlights the invasion characteristics of *T. minuta* and its relationship with elevation in southeastern Xizang, offering valuable data for the ecological management of invasive plant species in plateau regions.

## Introduction

Invasive plants refer to those plant species that are introduced intentionally or unintentionally into new ecosystems and reproduce and spread rapidly in the local area, thereby negatively affecting local biodiversity and ecosystem functions (Xiang et al., 2002; Wang et al., 2022; Qiang et al., 2010; Li et al., 2022). These plants usually have strong adaptability, reproductive capacity, and competitiveness, and can quickly occupy new ecological niches. They alter the ecological balance by outcompeting native species (Lei, Xiao & Feng, 2010). In recent years, with the intensification of global climate change and the increasing human activities, plant invasion has become increasingly common worldwide, becoming one of the key factors affecting ecosystem stability and biodiversity. Invasive plants profoundly affect the survival and distribution patterns of native species through various pathways, such as competing for resources, altering soil properties, and disrupting ecological processes. Therefore, studying the distribution characteristics of invasive plants under different ecological conditions and their impacts on the diversity of native biological communities is not only of great theoretical value but also of significant practical importance for ecological protection and species management.

*Tagetes minuta* is an annual herbaceous plant belonging to the genus *Tagetes* in the family *Asteraceae*. It is also known as stinking Roger (Xu et al., 2022), dwarf marigold, and fine-flowered marigold (Miao, Xu & Zhai, 2022). The plant can reach a height of up to 2.5 m (Zhang et al., 2014) and contains volatile oils that emit a strong aromatic odor. It is a typical invasive herbaceous species, native to South America (Zhang et al., 2019), and has now been widely distributed in many parts of the world (Yun et al., 2020). In China, *T. minuta* was first recorded as a naturalized species in the central mountainous area of Taichung City, Taiwan, in 2006 (Wang & Chen, 2006). The introduction pathway of *T. minuta* into China remains unclear, though experts speculate it was likely introduced *via* imported horticultural seedlings (Zhang et al., 2019). Since then, its distribution has gradually expanded and it has been successively found in several provinces in recent years, including Beijing, Hebei, Shandong, Jiangsu, Guangxi, and Xizang (Zhang et al., 2014; Yun et al., 2020; Dong et al., 2013; Xu & Tsering, 2015).

*T. minuta* has an extremely strong ability to reproduce and spread. Firstly, it produces a large number of seeds with high germination rates and strong dispersal capabilities, which can cover considerable distances (Zhang et al., 2014). In addition, *T. minuta* has a strong adaptability to habitats, and it can grow and spread rapidly in arid regions, saline-alkali lands, and infertile soils (Dong et al., 2013). Because of this, *T. minuta* has posed a threat to the ecological environment in many tropical and subtropical regions around the world (Singh, Singh & Kaul, 2003; Hulina, 2008). Although research on the invasive plant *T. minuta* in China is relatively limited, existing studies have mainly focused on reports of species naturalization (Wang & Chen, 2006; Qiu et al., 2020; Hulina, 2008), morphological characteristics, the harm it causes, and the impact of its root system on soil microbial diversity (Yun et al., 2020). Zhang et al. (2019) assessed its ecological risks, while Tu et al. (2018) conducted competition experiments between *T. minuta* and *Hordeum vulgare* var. *trifurcatum* and carried out field research. The results showed that the invasion of *T. minuta* can significantly increase the mortality rate of *H.vulgare* var. *trifurcatum*. Moreover, internationally, research has mostly focused on the chemical composition of *T. minuta* essential oil and its resource utilization (Eguaras et al., 2005), the leaves and flowers of *T. minuta* accumulate a rich profile of essential oils and can be used in medicines, condiments, foods, cosmetics, and biocides (Wang et al., 2023). In countries such as South Africa and India, *T. minuta* is actively exploited as a botanical source of insecticidal, microbicidal and repellent agents (Cornelius & Wycliffe, 2016).

Southeastern Tibet serves as a hotspot region on the Qinghai-Tibet Plateau, boasting the highest richness of endemic species (Yu et al., 2018). In the section from Nyingchi to Shannan along the Yarlung Zangbo River in southeastern Xizang, *T. minuta* has become a seriously invasive plant species and has the potential to further spread into the original habitats. The ecological environment of the Tibet is highly fragile. In the event of a large-scale invasion of invasive species, the ecological consequences would be disastrous (Xu et al., 2022). However, there is currently a lack of systematic research based on field plant community surveys regarding the specific impacts of *T. minuta* on community species diversity distribution patterns and invasion dynamics under different elevation gradients. Variations in elevation can, to a certain extent, influence the temperature, humidity, and light intensity of a specific region. Consequently, elevation change has become one of the crucial factors affecting species diversity (Yuan et al., 2024). Although previous studies have explored the impacts of elevation on native plants and some invasive species, research on the invasion dynamics in southeastern Tibet (Li, Ni & Zhao, 2023), particularly in the context of an elevation gradient, remains inadequate. Therefore, this study aims to systematically investigate the species diversity distribution characteristics of the *T. minuta*-invaded plant communities under an elevation gradient in the section from Nyingchi to Shannan along the Yarlung Zangbo River. It also aims to explore its invasion dynamics in southeastern Xizang, including characteristics such as the height, abundance, and cover of *T. minuta*. The results of this study are intended to provide a scientific basis for the ecological control of *T. minuta* and theoretical support for ecological protection and management of invasive alien species in the region.

## Study Area and Methods

### Overview of the study area

**Figure 1:**
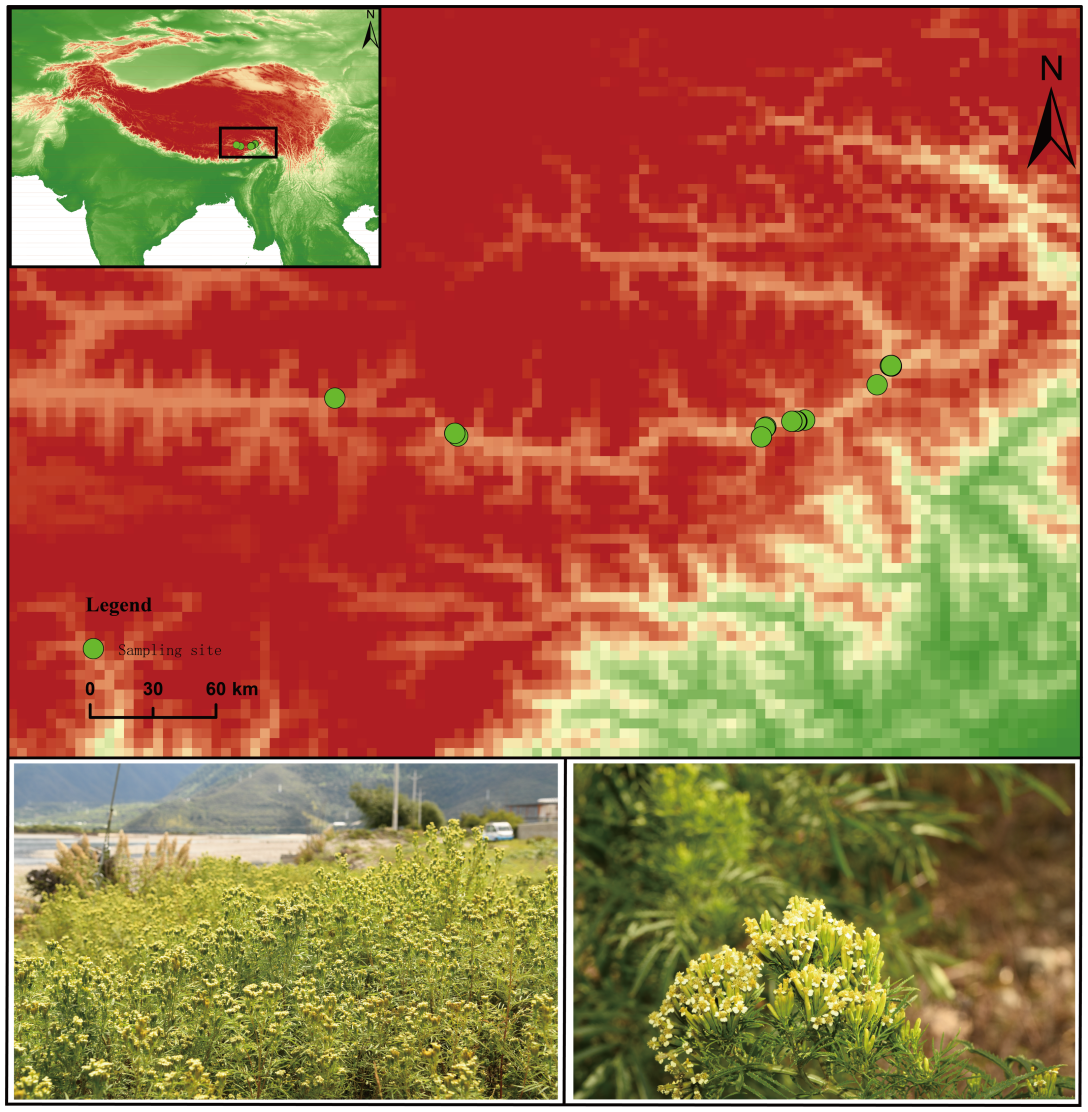
Overview map of the study area.

The southeastern Qinghai–Tibet Plateau encompasses the eastern Himalaya and the Hengduan Mountains, one of the world’s recognized biodiversity hotspots and a critical ecological security barrier for the plateau (Wan et al., 2024). Our study area is located in the section from Nyingchi to Shannan along the Yarlung Zangbo River in southeastern Xizang (29°07′28.16″N–29°25′50.38″N, 92°04′13.02″E–94°27′27.53″E, elevation 2,925–3,553 m) (Fig. 1). The area has a plateau temperate semi-humid monsoon climate (Qiu et al., 2020), with an annual precipitation of 350–641 mm, an average annual temperature of 8.2–11.0 °C, an average annual sunshine duration of 2000–2500 h, and a frost-free period of 130–170 days (Qiu et al., 2021). Owing to substantial elevational gradient, complex topographic and edaphic conditions (Qiu, 2008), and marked spatial heterogeneity in hydro-thermal regimes, the region harbors exceptional floristic diversity. It hosts the largest remaining tracts of primary forest on the Qinghai-Tibet Plateau, dominated by cold-temperate coniferous communities (Cheng, Tian & Zhou, 2022).

### Methods

This study focuses on the plant communities in the invaded areas of *T. minuta*. The survey was conducted using the quadrat method. When establishing sampling points, the distribution of *T. minuta* across different elevations and habitat types was taken into account to ensure representative sampling of the study area’s ecological characteristics. A total of 31 invaded quadrats were surveyed, each measuring 2 m × 2 m. The habitat types included nine sites along national highways near residential areas (GJ), five sites at garbage dump locations (LJ), four sites at artificial tree pits (soil pits dug for planting street trees) along national highways (GH), four sites at drainage ditches along national highways (GP), three sites next to abandoned buildings (FJ), three sites at riverbank sandy areas (HB), and three sites at construction sites (JZ). Within each quadrat, the species names of plants, the abundance, cover, and height of *T. minuta* were investigated and recorded. Additionally, geographical coordinates, vegetation types, and habitat characteristics, including longitude, latitude, elevation, and habitat type, were documented. These data were used to analyze the species composition structure and diversity characteristics of *T. minuta* communities. For plants that were difficult to identify in the field, specimens were collected and brought back to the laboratory for detailed identification.

### Data processing

Data analysis was performed using Microsoft Office Excel 2010 (Microsoft, Redmond, WA, USA) and R v4.3.3 software (R Core Team, 2023) for data organization and calculation of diversity indices (vegan package). The Euclidean distance matrix was calculated based on species abundance to measure the dissimilarity between quadrats. The complete linkage clustering method (stats package) was used to classify the 31 community quadrats into three clusters. One-way analysis of variance (ANOVA) and the Least Significant Difference (LSD) method were applied to conduct variance analysis and multiple comparisons of the diversity indices among different clusters and the characteristic variations of *T. minuta* in different vegetation types and habitat types. The results were visualized using Origin 2021 (OriginLab, Northampton, MA, USA).

The species diversity within the communities was measured using species richness (*R*), the Shannon-Weiner index (*H′*), the Simpson index (*D*), and the Pielou evenness index (*J*) (Tong et al., 2024). The calculation formulas are as follows:

Shannon-Weiner Index (H′):

(1) \begin{eqnarray*}\begin{array}{@{}c@{}} \displaystyle H{^{\prime}}=-\sum _{i=1}^{S}\,{P}_{i}\ln \nolimits ~ \left( {P}_{i} \right) \end{array}.\end{eqnarray*}

Simpson Index (*D*):

(2) \begin{eqnarray*}\begin{array}{@{}c@{}} \displaystyle D=1-\sum _{i=1}^{S}\,{P}_{i}^{2},{P}_{i}= \frac{{N}_{i}}{N} \end{array}.\end{eqnarray*}

Pielou’s Evenness Index (*J*)

(3) \begin{eqnarray*}\begin{array}{@{}c@{}} \displaystyle J= \frac{H}{\ln \nolimits \left( S \right) } \end{array}.\end{eqnarray*}

Species Richness (*R*)

(4) \begin{eqnarray*}\begin{array}{@{}c@{}} \displaystyle R=\mathrm{S} \end{array}.\end{eqnarray*}

In the formulas, *S* represents the number of species within the quadrat, *N* denotes the total number of individuals of all species in the quadrat, *N*_*i*_ is the number of individuals of the *i*-th species, and *P*_*i*_ is the proportion of individuals of species *i* to the total number of individuals.

## Results and Analysis

### Species domposition of *Tagetes minuta* invaded communities

A total of 78 plant species belonging to 28 families and 69 genera were found in the 31 community quadrats (Table 1). The family with the highest number of species was Asteraceae, with 19 species, accounting for 24% of the total species in the invaded areas. Poaceae and Rosaceae followed, each with nine species, representing 12% of the total species. The Fabaceae family had seven species. When classified by life form, herbaceous plants were dominant, with few woody and shrub species. There were 31 annual and biennial species and 47 perennial species. In addition to *T. minuta*, nine other invasive alien plants were found in the invaded communities, such as *Sonchus oleraceus*, *Galinsoga parviflora*, *Senecio vulgaris*, and *Erigeron canadensis*, accounting for 12% of all plants in the invaded areas.

**Table 1:** The species composition of the community invaded by *Tagetes minuta*.

Family	Number of genera	Percentage of genera (%)	Number of species	Percentage of species (%)
Asteraceae	14	20.291	19	24.359
Poaceae	9	13.043	9	11.538
Rosaceae	7	10.145	9	11.538
Fabaceae	6	8.697	7	8.975
Lamceiaae	3	4.348	3	3.847
Polygonaceae	3	4.348	3	3.847
Brassicaceae	2	2.899	2	2.564
Solanaceae	2	2.899	2	2.564
Scrophulariaceae	2	2.899	2	2.564
Rubiaceae	2	2.899	2	2.564
Boraginaceae	2	2.899	2	2.564
Euphorbiaceae	1	1.449	2	2.564
Oleaceae	1	1.449	1	1.282
Equisetaceae	1	1.449	1	1.282
Violaceae	1	1.449	1	1.282
Malvaceae	1	1.449	1	1.282
Campanulaceae	1	1.449	1	1.282
Onagraceae	1	1.449	1	1.282
Gentianaceae	1	1.449	1	1.282
Geraniaceae	1	1.449	1	1.282
Ranunculaceae	1	1.449	1	1.282
Cyperaceae	1	1.449	1	1.282
Polypodiaceae	1	1.449	1	1.282
Mazaceae	1	1.449	1	1.282
Adoxaceae	1	1.449	1	1.282
Amaranthaceae	1	1.449	1	1.282
Oxalidaceae	1	1.449	1	1.282
Plantaginaceae	1	1.449	1	1.282
Total	69	100	78	100

At the family level, in the plant communities invaded by *T. minuta*, there are four families with more than three species. These families account for only 14% of the total number of families but make up 56% of the total species number. There are eight families with two to three species, such as Lamiaceae (three species), Polygonaceae (three species), Scrophulariaceae (two species), and Rubiaceae (two species). There are a total of 16 families with only one species, which is the largest number of families and accounts for 57% of the total number of families. Examples include Ranunculaceae, Cyperaceae, and Violaceae.

At the genus level, the genus with the highest number of species is *Artemisia* (four species). There are six genera with two species, accounting for 9% of the total number of genera and 15% of the total number of species, such as *Euphorbia*, *Astragalus*, and *Taraxacum*. The majority of genera, a total of 62, contain only one species, representing 90% of the total number of genera and 79% of the total number of species. Examples include *Plantago*, *Melilotus*, *Erodium*, and *Datura*.

In addition, species with relatively high frequency of occurrence in the plant communities of *T. minuta* include *Poa annua*, *Digitaria cruciata*, *Plantago depressa*, *Artemisia sieversiana*, *Eragrostis nigra*, *Setaria viridis*, and *Erodium cicutarium*.

### Cluster analysis of *Tagetes minuta* communities

**Figure 2:**
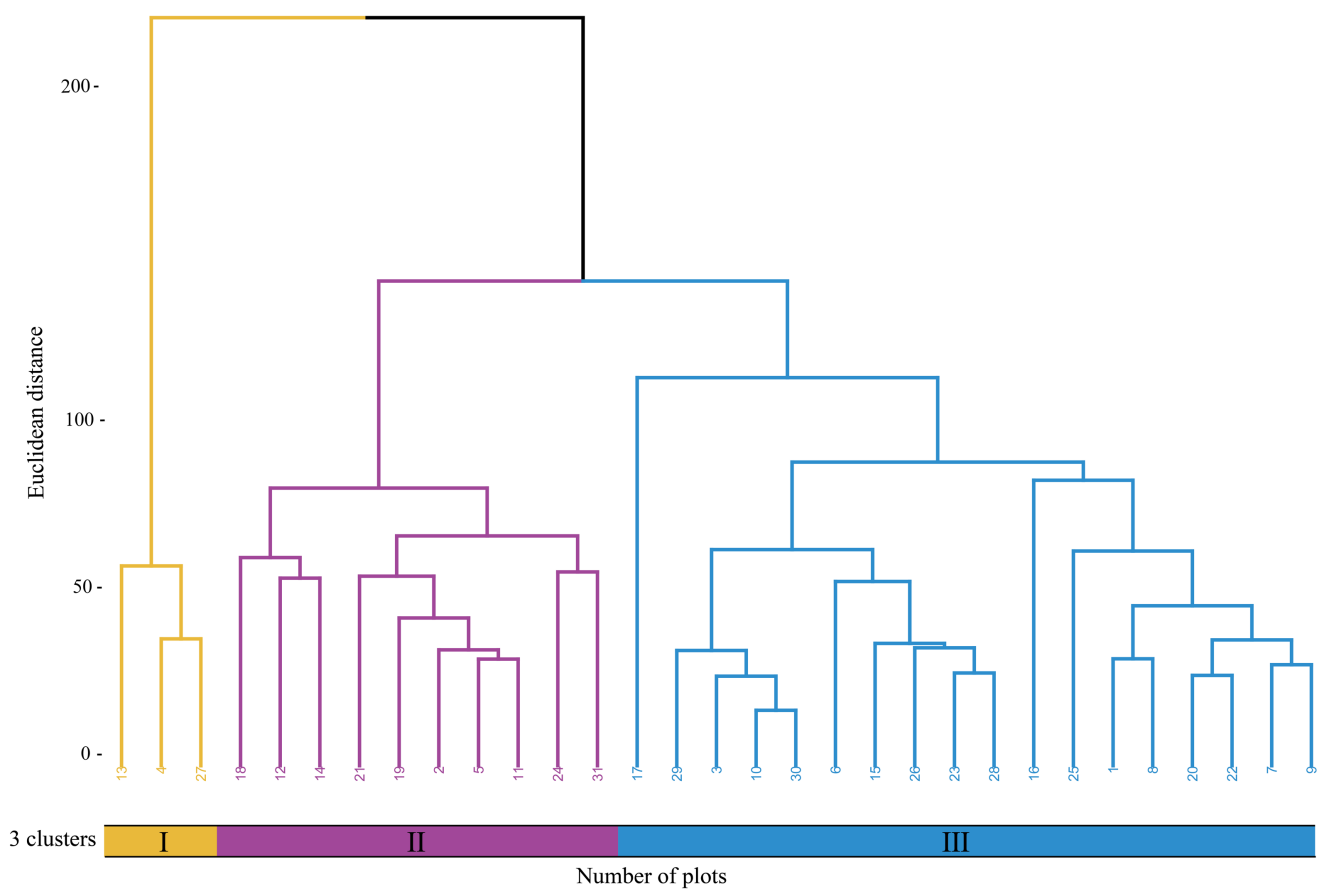
Cluster dendrogram of sample plots. Numerals I, II, and III represent three different plant communities.

According to the cluster analysis using the complete linkage method(Fig. 2), the communities dominated by *Poa annua* plus *Plantago depressa* (Cluster Group I) mainly includes habitat types such as national highway roadside residential areas, garbage dump sites, and areas next to abandoned buildings. The communities co-dominated by *Eragrotis Pilosa* and *Plantago depressa* (Cluster Group II) consists of habitat types like national highway roadside residential areas and roadside ditches. The communities dominated by *Poa annua* and *Digitaria cruciata* (Cluster Group III) includes national highway roadside residential areas, garbage dump sites, and riverbank sandy areas.

A comparison of the diversity indices among the three clusters (Table 2) reveals that Cluster Group II has a significantly higher Shannon-Wiener index than Cluster Group I (0.85) and Cluster Group III (1.16) (*P* < 0.05), indicating higher species diversity in Cluster Group II. The Simpson index, which measures the dominance concentration in a community, reflects the impact of different vegetation types on the evenness of species distribution. The Pielou evenness index indicates the evenness of the distribution of *T. minuta* within the community. Cluster Group II has significantly higher Shannon-Wiener index, Simpson diversity index, and Pielou evenness index than Cluster Groups I and III. In terms of species richness index, no significant differences were found among the three clusters.

**Table 2:** Comparison of community species diversity among different cluster groups.

Indices	Cluster Group		
	I	II	III
Shannon-Wiener index	0.85a	1.66b	1.16a
Simpson’s index	0.33a	0.69b	0.49a
Species Richness	10.67a	12.00a	10.22a
Pielou evenness index	0.36a	0.67b	0.50a

**Notes.**

a,b The significant different at 5% probability level in each row.

### Invasion characteristics of *Tagetes minuta* under different habitats

**Figure 3:**
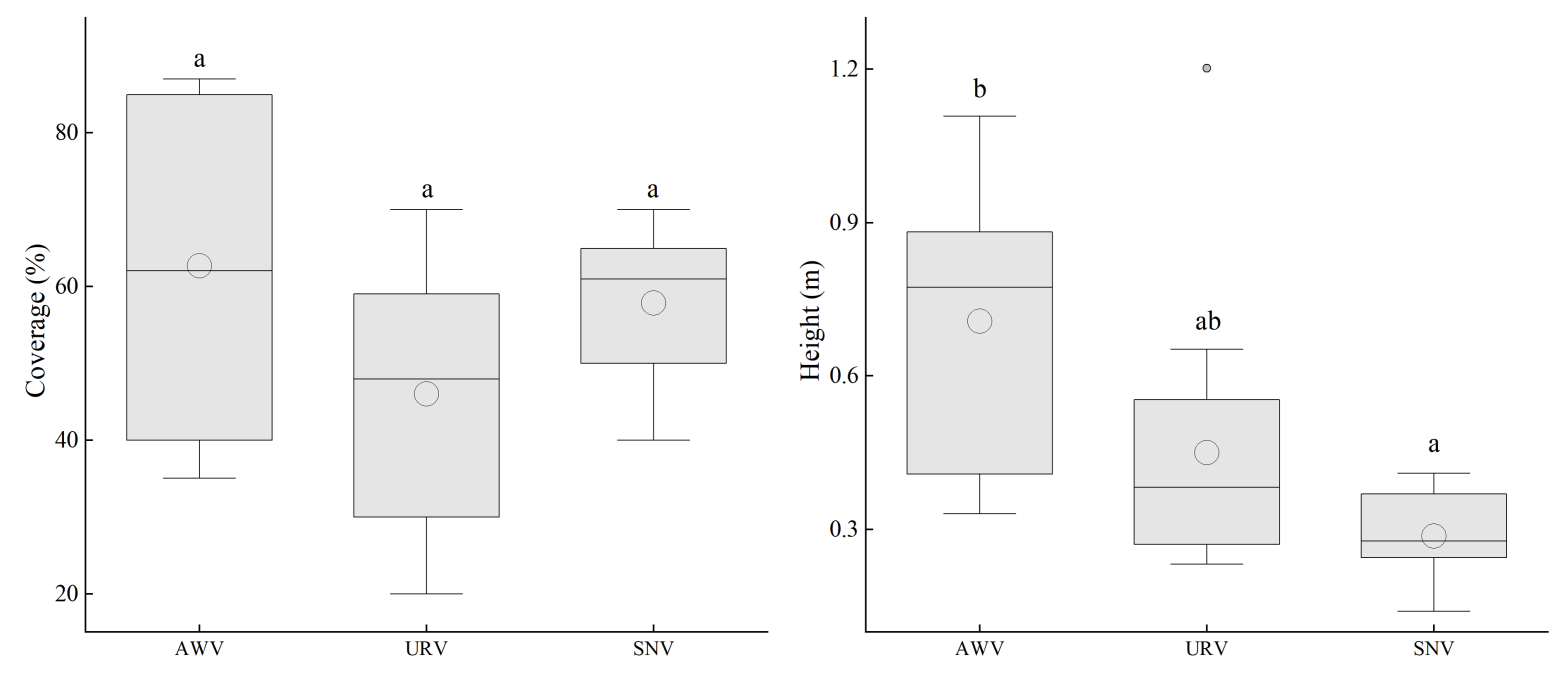
The cover and height of the invasive alien plant *Tagetes minuta* in different vegetation types. Different lowercase letters (*e.g.*, a, b) indicate significant differences among vegetation types (*p* < 0.05). AWV, Anthropogenic Wasted Vegetation; URV, Urban Road Vegetation; SNV, Semi-Natural Vegetation.

Under different vegetation types, there are significant differences in the height of *T. minuta*, while its cover does not show a significant correlation with vegetation types (Fig. 3). In terms of cover, it is shown that Abandoned Waste Vegetation (AWV) > Semi-Natural Vegetation (SNV) > Urban Roadside Vegetation (URV); in terms of height, the order is AWV > URV > SNV. In particular, in AWV, the cover and height of *T. minuta* are significantly higher than those in the other two types of vegetation. All three types of vegetation are invaded by *T. minuta* to varying degrees, with the situation being more severe in AWV.

**Figure 4:**
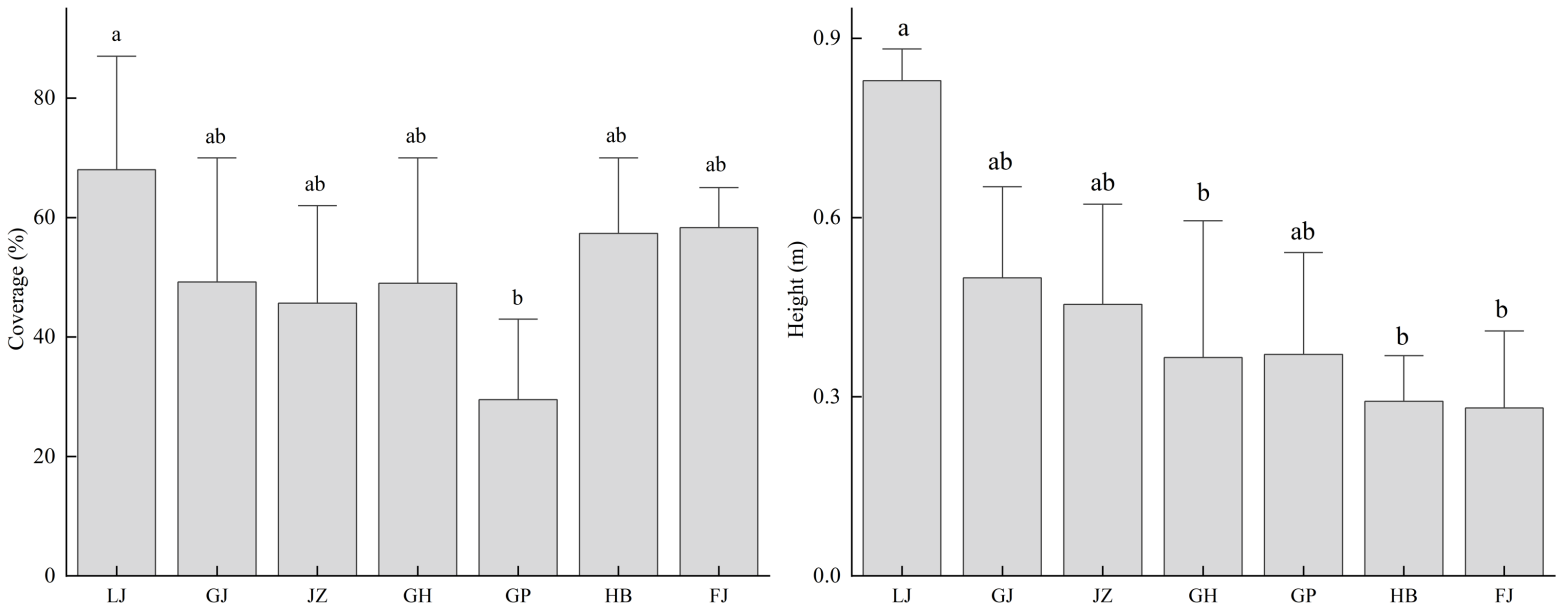
The relationship between different habitat types and the coverage and height of *Tagetes minuta*. Different lowercase letters (*e.g.*, a, b) indicate significant differences among habitat types.

By analyzing the growth conditions of *T. minuta* in different habitats, it was found that its cover and height show significant differences among the various habitats (Fig. 4). The cover of *T. minuta at* garbage dump sites is significantly higher than that at roadside ditches (*P* < 0.05); its height at garbage dump sites is significantly higher than that at artificial tree pits along national highways, riverbank sandy areas, and next to abandoned buildings (*P* < 0.05). Both the cover and height of *T. minuta* at garbage dump sites are the highest among all habitat types.

### Diversity patterns of *Tagetes minuta* communities along the altitudinal gradient

**Figure 5:**
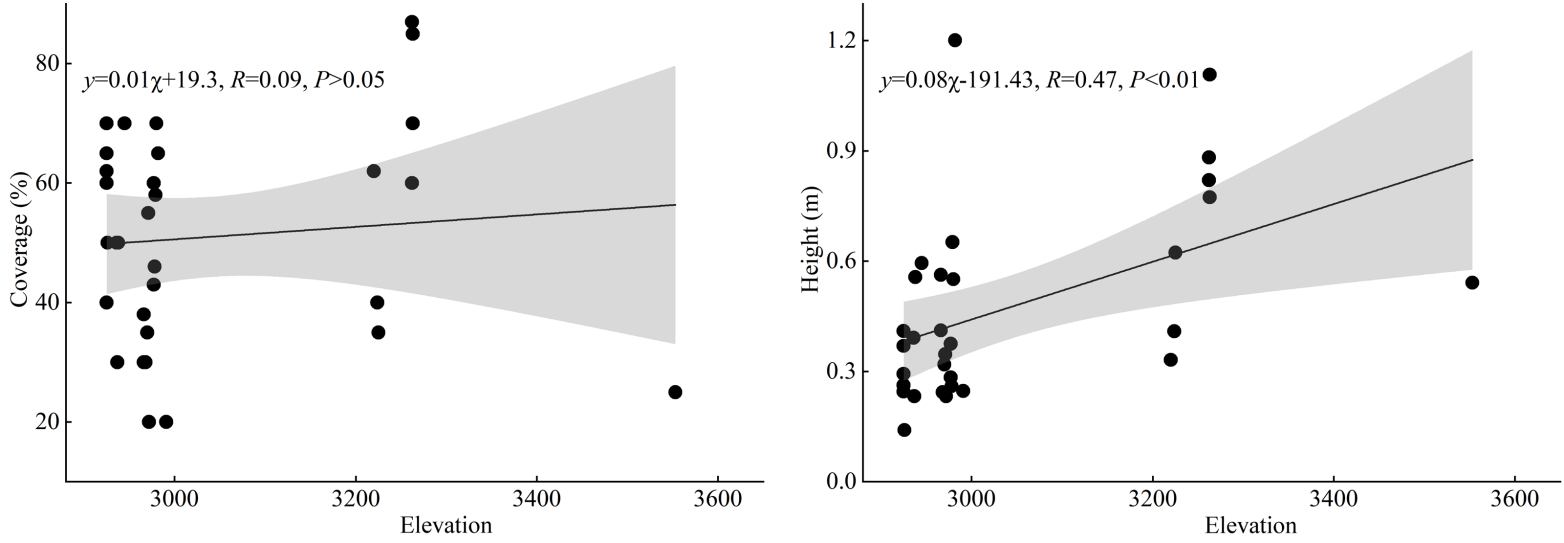
The correlation between the cover and height of *T. minuta* and the altitudinal gradient.

As illustrated in Fig. 5, the results of linear regression analysis indicate that there is no significant correlation between the cover of *T. minuta* and elevation (*P* > 0.05), suggesting that elevation has no significant effect on the cover of *T. minuta*. However, a highly significant positive correlation was found between the height of *T. minuta* and elevation (*P* < 0.01), indicating that the biomass growth of *T. minuta* in the height direction is less restricted by elevation.

**Figure 6:**
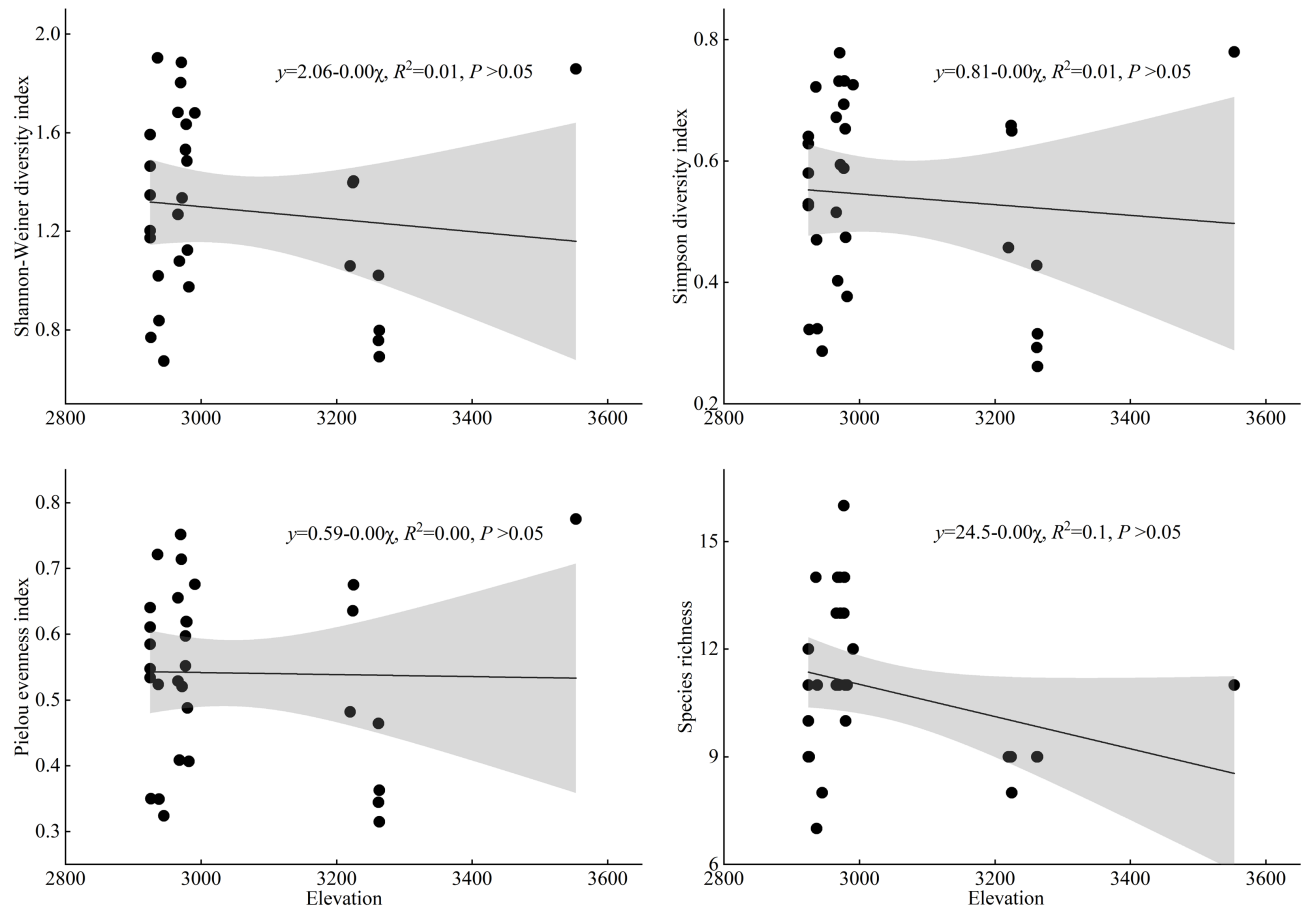
The changing trend of plant species diversity in *Tagetes minuta* communities with altitude.

As shown in Fig. 6, this study used linear regression to analyze the correlation between the species diversity indices of *T. minuta*-invaded plant communities and the altitudinal factor. The results show that there is no significant correlation between elevation and any of the diversity indices of *T. minuta*-invaded plant communities. This indicates that elevation has no significant effect on the species diversity of *T. minuta* communities.

## Discussion

### Species composition and distribution of *Tagetes minuta* communities

After arriving in a new habitat, invasive alien species can disrupt the functions and structure of ecosystems through allelopathy, competition, and other means (Gioria & Osborne, 2014), thereby breaking the original ecological balance of the ecosystem (Lei, Xiao & Feng, 2010). Research by Tu et al. (2018) found that the invasion of *T. minuta* significantly suppresses the survival of the local crop *H.vulgare* var. *trifurcatum*, markedly reducing its survival rate and demonstrating strong competitive and inhibitory abilities compared with indigenous crops. The results of our survey of *T. minuta* communities in southeastern Xizang showed that a total of 78 plant species, belonging to 28 families and 69 genera, were recorded. In these communities, Asteraceae, Poaceae, and Rosaceae were the dominant families, which is similar to the family distribution of some other common invasive plants. For example, in the communities of *Ageratina adenophora* (Jiang et al., 2024), *Datura stramonium* (Wang et al., 2024), and *G. parviflora* (He, Yang & Shi, 2020), plants of the family Asteraceae often hold important positions. This suggests that plants of these families may have certain advantages in adapting to the environment and in their ability to spread. In terms of life forms, perennial herbs were the most abundant in the *T. minuta* communities, which is consistent with the findings of Qi et al. (2022a) and Li et al. (2023) on the species composition of *Solidago canadensis* invaded communities. The second most abundant were annual herbs and biennial herbs. The coexistence of multiple invasive plants may lead to competition for resources among them, but it may also enhance their invasive capacity against local ecosystems through synergistic effects (Oduor, Yu & Liu, 2024).

### The impact of different habitats on the invasion characteristics of *Tagetes minuta*

As an invasive plant, this study used the height and cover of *T. minuta* as indicators of invasion characteristics to investigate its distribution features and influencing factors in different vegetation types in southeastern Xizang. The results showed that the cover and height of *T. minuta* varied among different vegetation types, with more severe invasion in abandoned waste vegetation. Ngondya & Munishi (2021) investigated the impact of the invasive alien plants *Gutenbergia cordifolia* and *T. minuta* on native plants in the Ngorongoro Crater, Tanzania. The results revealed that in areas with low invasion, the ground cover of native species was twice as high as that in moderately and highly invaded plots. Furthermore, the average height of native plants in highly invaded areas was double that of native plants in areas with low invasion. This indicates that *T. minuta* has stronger invasion ability and competitive advantage in habitats with higher human disturbance and weaker vegetation recovery (Qiu et al., 2021). Moreover, in different habitat types such as garbage dump sites, the cover and height of *T. minuta* were the highest among all habitat types, which may be related to factors such as soil fertility and human activities in these habitats. Wang et al. (2024) found that *D. stramonium* mainly grows in places with greater human disturbance, such as abandoned farmland, construction waste piles, domestic waste piles, and roadside areas, which is consistent with our research results. Qiu et al. (2021) also pointed out that *T. minuta* has a larger biomass in habitats such as roadsides and wastelands, and its phenotypic plasticity is higher, allowing it to adjust the biomass of various components to adapt to different environmental conditions. This adaptability enables *T. minuta* to successfully invade different vegetation types.

Through the study of species diversity of *T. minuta* in various habitats, it was found that the type and degree of human disturbance, as well as habitat type, are the factors that determine the invasion characteristics of *T. minuta*. The Shannon-Wiener index and Simpson index of Cluster Group II are significantly higher than those of Cluster Groups I and III, indicating that this cluster has higher species diversity and a more even distribution of species. This may be related to the higher disturbance frequency of the habitat types included in Cluster Group II. The main habitat types in Cluster Group II include national highway roadside residential areas, national highway roadside ditches, garbage dump sites, areas next to abandoned buildings, and construction sites, which dominate in Cluster Group II. Frequent human activities may have altered the structure of the communities, providing suitable niches for the invasion of *T. minuta* and also promoting an increase in species diversity. However, in terms of the species richness index, no significant differences were found among the three clusters, indicating that the invasion of *T. minuta* seems to have relatively small impact on species richness but a more significant impact on the evenness of species distribution.

These findings are consistent with previous research results, which indicate that disturbance in the habitat environment is one of the main causes of the establishment of alien species (Hertling & Lubke, 2000). Such disturbances lead to a rapid increase in resources in the short term, providing opportunities for alien species that can quickly exploit these resources to expand rapidly (Hertling & Lubke, 2000). At the same time, human activities can increase the propagule pressure in disturbed habitats, as travelers bring in large numbers of seeds through their clothing, shoes, or vehicles, and the seeds of almost all roadside plants may be introduced into new habitats by humans (Mack & Lonsdale, 2001; Schmidt, 1989). Therefore, areas with more frequent human activities are often more likely to have a greater number of alien species. This can explain the trend of high species diversity of *T. minuta* in habitats such as national highway roadside residential areas and garbage dump sites. In addition, roads, as landscape corridors, not only increase edge effects but also promote the invasion of alien species (Zhou, Peng & Lin, 2009). Studies have shown that roads play an important role in the spread of alien species to adjacent habitats (such as forests) (Nelson Cara, Halpern & Agee, 2008). These findings are in line with the results of this study, that is, stronger human disturbance can promote the occurrence of plant invasions. In specific habitats, such as garbage dump sites and national highway roadside residential areas, human activities are frequent and human disturbance is strong, which provides favorable conditions for invasive plants. The invasion characteristics of these habitats are consistent with the research results of Li et al. (2018). Areas with frequent human activities are more susceptible to invasion by alien species, and the vacant niches in these areas provide convenience for the colonization and spread of invasive species.

### The diversity pattern of community invaded by tagetes patula

The study of the mechanisms of alien plant invasion and their influencing factors has always been a hot topic in ecology (Yang et al., 2011; Jiang et al., 2024; Wang et al., 2024). Research shows that the degree of alien plant invasion is influenced by a variety of factors, including the ecological interactions between the invaded area and the local plant community, as well as the impact of human activities (Li, Ni & Zhao, 2023; Wang, Bai & Sang, 2017). Changes in hydrothermal conditions caused by factors such as elevation are important indicators in the study of species diversity gradient (Hao et al., 2001; Zhang et al., 2021). As elevation increases, temperature decreases, atmospheric pressure and CO_2_ partial pressure drop, and light intensity increases, which can cause significant changes in the ecological and physiological characteristics of plants and may affect the distribution of plant species along the altitudinal gradient and the structure and composition of plant communities (Pan et al., 2009). elevation changes are decisive factors in the distribution and composition of plant community species (Guo et al., 2003).

Firstly, in terms of the invasion characteristics of *T. minuta*, linear regression analysis showed that there was no significant correlation between its cover and the elevation of the sampling points (*P* > 0.05), indicating that elevation did not limit the cover of *T. minuta*. This result may imply that the cover of *T. minuta* is influenced by a combination of various factors, and elevation is not the dominant factor across the elevational range studied here. However, there was a highly significant positive correlation between the height of *T. minuta* and the elevation of the sampling points (*P* < 0.01), which may be related to the specific environmental conditions in high-elevation areas, such as lower temperatures and stronger light, which may be conducive to the growth and development of *T. minuta*, allowing it to reach greater heights. Chen et al. (2022) showed that, with the increase of the altitudinal gradient, the population cover of *Galinsoga quadriradiata* did not show significant changes, but its plant height decreased significantly. This result is consistent with the findings of the present study regarding cover, but is opposite to the results of the present study in terms of the relationship between elevation and plant height. The altitudinal gradient in their study was approximately three times longer than that in the present study. This higher elevation, shorter stature result may primarily stem from the difference in gradient length. The harsh environmental conditions in high-elevation regions may reduce interspecific competition among plants. This potentially allows *T. minuta* greater access to resources (*e.g.*, light, water, and nutrients), thereby possibly enhancing its growth.

Secondly, regarding the relationship between the species diversity of the community invaded by *T. minuta* and the elevation factor, the results of the linear regression analysis showed that the Shannon-Weiner diversity index, Simpson diversity index, Pielou evenness index, and species richness index of the community invaded by *T. minuta* were not significantly correlated with elevation (*P* > 0.05). elevation had no significant effect on the species diversity of the community invaded by *T. minuta* (*P* > 0.05), and its diversity changes may be mainly regulated by other non-elevation factors. Qiu et al. (2010) predicted the potential distribution of the invasive plant *Mikania micrantha* in Guangzhou and found that elevation had a significant impact on the distribution of *Mikania micrantha*, which is contrary to the results of this study. One limitation of our study is the narrow elevation range covered. If our study had included a broader elevation range, we might have observed different patterns, particularly in plant height. Future studies covering a wider elevation range would help to gain a clearer understanding of these patterns.

Overall, the invasion characteristics of *T. minuta* and its impact on species diversity are regulated by a combination of various factors, including habitat type, degree of human disturbance, and environmental conditions. It exhibits a stronger invasion ability in habitats with frequent human disturbance, and the positive correlation between its height and elevation indicates that the specific environmental conditions in high-elevation areas may be conducive to its growth. However, the lack of a significant correlation between the species diversity of the community invaded by *T. minuta* and elevation suggests that its diversity changes may be more influenced by other non-altitudinal factors. The invasion of *T. minuta* poses a significant threat to the ecological environment (Qi et al., 2022a; Qi et al., 2022b). Therefore, it is necessary to conduct more in-depth research on the interactions among these factors to better understand the invasion mechanisms of *T. minuta* and to provide a scientific basis for implementing appropriate control measures and ecological security construction. Furthermore, future studies should specifically investigate the ecological responses and invasive potential of *Tagetes minuta* across a broader elevation gradient and diverse climatic zones, as these environmental variables may significantly influence its adaptation strategies and competitive interactions with native species. Such research would enhance our understanding of the mechanisms driving its invasion under varying conditions and inform region-specific management approaches to mitigate its ecological risks.

## Conclusion

This study investigated the invasion characteristics of *T. minuta* in the southeastern Xizang Yarlung Zangbo River Basin through field surveys. The results showed that: (1) the main associated species in the invaded areas of *T. minuta* are plants from the families Asteraceae, Poaceae, and Rosaceae; (2) abandoned human-impacted vegetation, urban roadside vegetation, and semi-natural vegetation were all subject to varying degrees of invasion by *T. minuta*, with the invasion being more severe in abandoned human-impacted vegetation. Rubbish dump sites were identified as the key habitat types invaded by *T. minuta*; (3) the height of *T. minuta* was significantly positively correlated with elevation, while its cover showed no significant correlation with elevation; (4) moreover, the species diversity indices of the invaded communities were not significantly correlated with elevation, indicating that the invasion characteristics of *T. minuta* and its impact on local biodiversity are mainly driven by human activities and habitat types, rather than the influence of the elevation gradient.

##  Supplemental Information

10.7717/peerj.20573/supp-1 Supplemental Information 1 Species Composition

10.7717/peerj.20573/supp-2 Supplemental Information 2 Original species data from quadrats

10.7717/peerj.20573/supp-3 Supplemental Information 3 Geographical location and habitat type information

## Additional Information and Declarations

### Data Availability

The following information was supplied regarding data availability: The raw data is available in the Supplemental Files.
